# Audiological Profile of Patients with Vitiligo

**DOI:** 10.1055/s-0045-1809648

**Published:** 2025-10-16

**Authors:** Inna Fayaz, Neha Saraf, Gopika Kalsotra, Parmod Kalsotra, Vikas Mahajan, Samreen Dilawar, Sahil Kalsotra

**Affiliations:** 1 Department of Ear, Nose, and Throat – Head and Neck Surgery, Sri Maharaja Gulab Singh (SMGS) Hospital, Government Medical College, Jammu, Jammu and Kashmir, India; 2 Department of Anatomy, Hinduhridaysamrat Balasaheb Thackeray (HBT) Medical College, Dr. Rustom Narsi Cooper Municipal General Hospital, Mumbai, Maharashtra, India; 3 Department of Pediatrics, Acharya Shri Chander College of Medical Sciences (ASCOMS), Jammu, Jammu and Kashmir, India; 4 Department of Dermatology, Sri Maharaja Gulab Singh (SMGS) Hospital, Government Medical College, Jammu, Jammu and Kashmir, India; 5 Emergency Department, Shri Durga Dass Memorial (SDDM) Hospital, Jammu, Jammu and Kashmir, India

**Keywords:** vitiligo, SNHL, audiometry, ABR

## Abstract

**Introduction:**

Vitiligo is one of the most common causes of hypopigmentation; it occurs when melanin production is impaired due to the loss of melanocytes, which are present throughout the skin, in the epidermis, and in hair follicles, and are also seen in the mucosa, uvea, and several structures of the inner ear. Scientists have investigated the association between vitiligo and sensorineural hearing loss (SNHL); some believe there is a connection, while others disagree.

**Objective:**

To evaluate the relationship between vitiligo and SNHL.

**Methods:**

The present observational study was conducted from February 2023 to January 2024. A total of 54 patients with clinically diagnosed vitiligo were assessed for hearing impairment. Simultaneously, 50 healthy volunteers were included as a control group, and the data was analyzed to assess any potential association between vitiligo and SNHL.

**Results:**

The mean age of the control group was of 37.5(± 10) years, and that of the patients with vitiligo was of 38.8(± 11) years, and the male-to-female ratio was of 1:1. We observed SNHL in 32 (59.3%) patients with vitiligo, and those with vitiligo vulgaris were the most afflicted. The hearing loss was mild in 2 ears (3.1%), moderate in 24 (37.5%), and severe in 29 (45.3%), with 29 patients belonging to the group with high frequency hearing loss.

**Conclusion:**

Based on the findings; it is imperative that patients with vitiligo undergo routine auditory function assessments to facilitate the early detection of SNHL.

## Introduction

Vitiligo is a chronic immune-mediated disorder that causes various areas of the skin to lose the pigment that imparts it with color; the condition manifests in a variety of patterns, ranging from minute macules with scalloped borders to nearly whole-body depigmentation. Regardless of nationality or color, the illness affects ∼ 1 to 2% of the global population; the Indian subcontinent presents the highest incidence, followed by countries such as Mexico and Japan. 1 2 There are different theories about the pathogenesis of vitiligo, but the exact etiology is still unknown In genetically-predisposed individuals, autoimmune, cytotoxic, biochemical, oxidant-antioxidant, viral, and neurological pathways typically contribute to the degradation of melanocyte function. Patients with vitiligo and their first-degree relatives are more likely to present autoimmune disorders, including diabetes, thyroiditis, Grave's disease, Addison's disease, alopecia areata, and pernicious anemia. 3

Vitiligo can appear at any age, but its onset is often observed in childhood or early adulthood, with a peak in patients aged between 10 and 30 years. All races are equally affected, and equal vulnerability is observed in both genders. The susceptibility among female subjects is higher due to the use of cosmetics. 4 5 6 The onset of vitiligo is early in patients with a positive family history. 7 Stress, autoimmunity, mutations, melatonin receptor malfunction, and reduced proliferation and migration of melanocytes have all been linked to vitiligo. Additionally, the buildup of hazardous intermediate products of melanin synthesis has been linked to it. 8

The melanocyte lineage is derived from the neural crest and melanocytes are present in various organs, including the epidermis, the hair bulbs of the follicles, the uveal tract and the pigment epithelium of the retina, the inner ear, and the leptomeninges. 9 In 1831, Corti was the first to mention the presence of pigmented cells in the inner ear. 10 Melanocytes are most commonly found in the cochlear section of the inner ear, as well as the modiolus, osseous spiral lamina, Reissner's membrane, and vascular stria. 11 Studies describing the distribution of melanocytes in the vestibule are scarce, but those conducted to date have found their presence in the vestibular labyrinth, including the utricle, saccule, pars commune, ampulla, endolymphatic duct, and sac 1 .

The precise role of melanocytes in the inner ear is uncertain; however, it is believed that they contribute to vasomotor activity. 12 The most common sign of vitiligo is the discoloration of the skin; however, the pigment cells in other parts of the body might also be affected. 13

Certain studies 14 have reported auditory dysfunction in a few patients with vitiligo, although there is disagreement among various researchers in the literature. Therefore, the current study was performed in the Department of Ear, Nose, and Throat (ENT) – Head and Neck Surgery, as well as in the Department of Dermatology, of the Sri Maharaja Gulab Singh (SMGS) Hospital, Government Medical College, Jammu, India, to evaluate the auditory function of vitiligo patients.

## Methods

The current prospective, observational research was conducted at a tertiary care facility from February 2023 to January 2024, with 54 patients with clinically diagnosed vitiligo who were referred from the Dermatology Outpatient Department (OPD). Following permission from the institutional Ethics Committee and the obtainment of informed consent, all subjects were assessed for hearing loss through pure tone audiometry and brainstem evoked response audiometry (BERA). The data was then evaluated for any sensorineural hearing loss (SNHL) component. A control group consisting of 50 healthy volunteers was also included, matched for age and gender, to compare the findings. Any correlation between vitiligo and hearing loss was assessed in compliance with ethical standards.

The inclusion criteria were male and female patients with vitiligo of different pathogeneses (type, site, duration) aged between 20 and 60 years. The exclusion criteria were patients with a history of middle-ear disease or of head trauma, those with a familial history of hearing disability, subjects using ototoxic drugs, those with chronic exposure to noise, patients with systemic diseases such as diabetes mellitus and hypertension, and aged > 60 years (because age-related SNHL could be present above this age).

The case group was divided according to the clinical type of vitiligo (localized: focal, segmental or mucosal; generalized: acrofacial, vulgaris or mixed; and universal), the duration (> 18 months or < 18 months), and the progression of the disease (stable or unstable).

The study involved all participants undergoing a comprehensive review of their medical and otologic history, a general examination of the ENT areas, and a basic audiological assessment that included pure tone audiometry and auditory brainstem response (ABR).

As for the pure tone audiometry, following an otoscopic examination of the ear canal, the individuals were assessed audiometrically using the Audiolite diagnostic audiometer (Labat Asia Private Limited). Air conduction was tested at 0.25, 0.5, 1.0, 2.0, 4.0, and 8.0 KHz, as well as bone conduction at frequencies ranging from 0.25 KHz to 4.0 KHz. The pure tone averages (PTAs) were determined using the frequencies of 0.5, 1, 2, and 4 KHz.

Regarding the ABR, we examined the absolute latency values of waves I, III, and V and the interpeak latency values of waves I-III, III-V, and I-V in both ears. The ABR measurements were conducted using the ICS Charter EP 200 equipment (GN Otometrics). Three electrodes were installed: one on the ipsilateral mastoid (inverted), one on the vertex (non-inverted), and one on the contralateral mastoid (which acted as the ground electrode). The interelectrode impedance was maintained at 5 kΩ. TDH 39 headphones (Telephonics Corporation) emitted rarefaction clicks at a frequency of 13.1 clicks per second, with an intensity measured at 80 dB normal hearing level (nHL), and each click had a duration of 0.1 milliseconds (ms). The screen's analysis time was set at 10 ms.

## Results

The current study included 54 subjects with clinically diagnosed vitiligo (108 ears) and a control group of 50 healthy volunteers (100 ears). The vitiligo group consisted of an equal number of male (27) and female (27) patients, maintaining a male-to-female ratio of 1:1. The control group was composed of 27 male and 23 female participants. The mean age of the vitiligo group was of 38.8 ± 11 (range: 20–61) years, and that of the control group was of 37.5 ± 10 (range: 22–60) years. The mean disease duration in the vitiligo group was of 15.9 ± 11.5 (range: 1–47) years. In total, 32 patients (59.3%) with vitiligo presented SNHL on pure tone audiometry, and the highest prevalence was found in the age group between 36 and 50 years, with 19 patients (35.2%), followed by 9 patients (16.7%) aged between 20 and 35 years, and 4 (7.4%) aged between 51 and 60 years, as shown in Table 1 .

**Table 1:** Sensorineural hearing loss (SNHL) in relation to age

Age group	With SNHL: n (%)	Without SNHL: n (%)	Total: n (%)	
**20–35 years**	9 (16.7%)	14 (25.9%)	23 (42.6%)	Chi-squared = 7.81; degrees of freedom = 2; *p* = 0.020
**36–50 years**	19 (35.2%)	5 (9.3%)	24 (44.4%)	
**51–60 years**	4 (7.4%)	3 (5.6%)	7 (13.%)	
**Total**	32 (59.3%)	22 (40.7%)	54 (100%)	

Among the 27 (50%) female subjects in the vitiligo group, 15 (27.8%) presented with SNHL, and most of them had the generalized type of vitiligo, either vitiligo vulgaris or acrofacial. Among the 27 (50%) male subjects, 10 (31.5%) presented with SNHL, and most had the acrofacial type, followed by vitiligo vulgaris, as shown in Table 2 .

**Table 2:** Sensorineural hearing loss (SNHL) in relation to gender

Gender	With SNHL: n (%)	Without SNHL: n (%)	Total: n (%)	*p* = 0.58
Female	15 (27.8%)	12 (22.2%)	27 (50%)	
Male	17 (31.5%)	10 (18.5%)	27 (50%)	
Total	32 (59.3%)	22 (40.7%)	54 (100%)	

The distribution of the vitiligo subtypes among the subjects who presented SNHL was as follows: 9 of the 19 (35.2%) patients with vitiligo vulgaris; 2 of the 3 (3.7%) patients with acral vitiligo; 7 of the 9 (13%) patients with focal vitiligo; 2 of the 4 (3.7%) patients with mucosal vitiligo; the single (1; 1.9%) patient with segmental vitiligo; and 8 of the 12 (14.8%) patients with acrofacial vitiligo, as shown in Table 3 .

**Table 3:** Sensorineural hearing loss (SNHL) in relation to vitiligo type

Type of vitiligo	With SNHL: n (%)	Without SNHL: n (%)	Total: n (%)	
**Acral**	2 (3.7%)	0 (0%)	2 (3.7%)	Chi-squared = 5.65; degrees of freedom = 6; *p* = 0.464
**Vulgaris**	9 (16.7%)	10 (18.5%)	19 (35.2%)	
**Focal**	7 (13%)	2 (3.7%)	9 (16.7%)	
**Mucosal**	2 (3.7%)	2 (3.7%)	4 (7.4%)	
**Segmental**	1 (1.9%)	0 (0%)	1 (1.9%)	
**Universalis**	3 (5.6%)	4 (7.4%)	7 (13%)	
**Acrofacial**	8 (14.8%)	4 (7.4%)	12 (22.2%)	
**Total**	32 (59.3%)	22 (40.7%)	54 (100%)	

No cases of SNHL were observed in the control group. The audiometric testing revealed normal hearing thresholds across all frequencies, and the BERA results showed typical waveforms, with no abnormalities in latency or interpeak latency.

On ABR, the mean absolute latencies for wave I were of 1.36 ± 0.366 ms (right) and 1.39 ± 0.373 ms (left); for wave III, they were of 3.54 ± 0.430 ms (right) and 3.73 ± 0.476 ms (left); and, wave V, they were of 5.92 ± 0.570 ms (right) and 5.75 ± 0.648 ms (left). The mean absolute interpeak latencies between waves I and III were of 2.24 ± 0.465 ms (right) and 2.35 ± 0.512 ms (left); between waves III and V, they were of 2.38 ± 0.562 ms (right) and 2.10 ± 0.749 ms (left); and between waves I and V, they were of 4.55 ± 0.548 (right) and 4.27 ± 0.731 (left), as shown in Table 4 . In most cases (23; 42.6%), the SNHL was bilateral, followed by 7 (13%) cases in the right ear and 2 (3.7%) in the left ear. In total, 22 (40.7%) patients did not present SNHL, as shown in Table 5 .

**Table 4:** ABR findings from patients with vitiligo

Ear/variable	Mean (ms)	SD (ms)	Minimum (ms)	Maximum (ma)
R/wave I latency	1.36	± 0.366	0.850	2.22
L/ wave I latency	1.39	± 0.373	1.010	2.96
R/wave III latency	3.54	± 0.430	2.880	4.66
L/ wave III latency	3.73	± 0.746	3.120	5.66
R/ wave V latency	5.92	± 0.570	4.920	6.80
L/ wave V latency	5.75	± 0.648	5.200	7.60
R/IPL of waves I–III	3.24	± 0.465	1.300	3.43
L/IPL of waves I–III	3.35	± 0.512	1.690	4.06
R/IPL of waves III–V	2.38	± 0.562	0.890	3.47
L/IPL of waves III–V	2.10	± 0.749	0.160	4.29
R/IPL of waves I–V	4.55	± 0.548	3.090	5.77
L/IPL of waves I–V	4.27	± 0.731	1.970	6.46

Abbreviations: ABR, auditory brainstem response; IPL, interpeak latency; L, left; ms, milliseconds; R, right; SD, standard deviation.

**Table 5:** Association between age groups and sensorineural hearing loss (SNHL)

Age group	Bilateral SNHL: n (%)	Unilateral SNHL – right ear: n (%)	Unilateral SNHL – left ear: n (%)	No SNHL: n (%)	Total: n (%)	
**20–35 years**	8 (14.8%)	1 (1.9%)	0 (0%)	14 (25.9%)	23 (42.6%)	Chi-squared = 12.8; degrees of freedom = 6; *p* = 0.047
**36–50 years**	11 (20.4%)	6 (11.1%)	2 (3.7%)	5 (9.3%)	24 (44.4%)	
**51–65 years**	4 (7.4%)	0 (0%)	0 (0%)	3 (5.6%)	7 (13%)	
**Total**	23 (42.6%)	7 (13%)	2 (3.7%)	22 (40.7%)	54 (100%)	

Among the controls, the mean absolute latencies for wave I were of 1.50 ± 0.15 ms (right) and 1.52 ± 0.17 ms (left), values consistent with the expected range of 1.5 ms to 0.2 ms. For wave III, the mean absolute latencies were of 3.50 ± 0.20 ms (right) and 3.55 ± 0.18 ms (left), which fall within the typical range of 3.5 ms to 0.3 ms. For wave V, the mean absolute latencies were of 5.70 ± 0.25 ms (right) and 5.75 ± 0.27 ms (left), in line with the expected range of 5.5 ms to 0.3 ms. The mean absolute interpeak latencies between waves I and III were of 2.00 ± 0.25 ms (right) and 2.03 ± 0.28 ms (left), within the expected range of 2.0 ms to 0.3 ms. The mean absolute interpeak latencies between waves III and V were of 2.20 ± 0.30 ms (right) and 2.22 ± 0.32 ms (left), within the typical range of 2.0 ms to 0.3 ms. Finally, the mean absolute interpeak latencies between waves I and V were of 4.20 ± 0.40 ms (right) and 4.25 ± 0.42 ms (left), corresponding to the typical range of 4.0 ms to 0.4 ms. None of controls presented SNHL. The audiometric testing revealed normal hearing thresholds, within the standard range, for all frequencies, and the BERA results showed typical waveforms and latencies, consistent with normal auditory function. These findings confirm the absence of any auditory impairment in the control group, with all 50 participants having normal hearing function and no evidence of SNHL in either ear. The hearing thresholds for pure tone audiometry were within the standard normal range, and all BERA results showed typical latencies and interpeak latencies, confirming the absence of auditory dysfunction.

Out of 30 patients with right-sided SNHL, the degree of impairment was severe on 15 (46.9%), moderate in 14 (43.8%), and in 1 (3.1%) subjects. These results were statistically significant, as shown in Table 6 . Out of 25 patients with left-sided SNHL, the degree of impairment was severe in 14 (43.8%), moderate in 10 (31.2%), and mild in 1 (3.1 %) subject. These results were statistically significant, as shown in Table 7 .

**Table 6:** Severity of the sensorineural hearing loss (SNHL) in the right ear

SNHL	Severe: n (%)	Moderate: n (%)	Mild: n (%)			Chi-squared = 46.4; degrees of freedom = 4; *p* < 0.001
	15 (46.9%)	14 (43.8%)	1 (3.1%)		

**Table 7:** Severity of the sensorineural hearing loss (SNHL) in the left ear

SNHL	Severe: n (%)	Moderate: n (%)	Mild: n (%)	*p* -value		
	14 (43.8%)	10 (31.2%)	1 (3.1%)	*p* <0.001		Chi-squared = 29.7; degrees of freedom = 3

Most patients (29; 90.6%) had high frequency (4,000 Hz, 6,000 Hz, and 8,000 Hz) hearing impairment, and only 3 (9.37%) had hearing impairment at lower frequencies.

There were no significant findings regarding the association of SNHL and the duration of vitiligo, as SNHL was observed in patients who had the condition for 1 year as well as more than 40 years. Moreover, all patients complained of the gradual progression of the disease; none of them had a stable disease throughout the years.

## Discussion

Vitiligo is an immune-mediated, hereditary disorder of depigmentation and occasionally genetic illness in which the cells that produce pigment are progressively lost. In vitiligo, whitish patches of various sizes and shapes appear on pigmented skin. In patients with vitiligo, the effects of the loss of melanin are mostly limited to the appearance of hypopigmentation in the skin; changes in extracutaneous locations have been recorded and occasionally associated to inner-ear dysfunction, causing hearing impairment 3 4 .

Apart from the skin, melanocytes are also present in vestibular organs, including the stria vascularis, and the cochlear modiolus. Their presence in the inner ear is essential for hair-cell survival because they perform a variety of functions, including maintaining homeostasis in the cochlea and stria vascularis, maintenance of endocochlear potential, and forming the balance between the endolymph and perilymph. 10 15 Thus, the current study was performed with the objective of analyzing the influence of vitiligo on hearing capabilities.

The present study included 54 patients diagnosed with vitiligo, with a female-to-male-ratio of 1:1 ages ranging from 20 to 60 years. Among these 54 patients, 32(59.3%) had SNHL, and most were aged between 36 and 50 years. Shankar et al. 16 found that vitiligo in patients aged ≥ 30 years was substantially related with aberrant auditory results. However, Sharma et al. 17 found no significant connection between age and SNHL in vitiligo patients; in their study, 32 patients (59.3%) developed SNHL, which was statistically significant. ( *p* < 0.05), with 9 patients (16.7%) presenting the vitiligo vulgaris type. These findings were comparable with those of Prabha et al. 18 In the present investigation, more female subjects presented SNHL compared to the male patients, which is in line with the findings of Sharifian et al. 19

In total, 23 (42.6%) individuals had bilateral SNHL, and 9 (16.7%), unilateral SNHL, with 7 (13%) cases of right ear involvement and 2 (3.7%) of left ear involvement in this study. In our investigation of 108 ears, most patients had severe SNHL (29 ears), followed by moderate SNHL (24 ears) and mild SNHL (2 ears); moreover, most cases were of high-frequency SNHL (4,000 Hz, 6,000 Hz, and 8,000 Hz). As observed in the current study and by Prabha et al, 18 vitiligo patients have hearing loss at high frequencies, indicating that the basal turn of the cochlea is likely affected.

There was no significant link found between SNHL and vitiligo duration, which is consistent with the findings by Mahdi et al. 20 The hypothesis that otic melanocytes are first affected by vitiligo before reaching a stable state may explain this observation.

For comparison, we included a control group of 50 healthy volunteers (100 ears), among whom no cases of SNHL were detected. The audiometric testing revealed normal hearing thresholds across all frequencies, and the BERA results showed typical waveforms, latencies, and interpeak latencies, consistent with normal auditory function. These findings in healthy volunteers were used as a baseline for comparison and provided a clear contrast to the abnormal auditory results observed in the vitiligo group. This comparison underscores the increased prevalence of SNHL, particularly at high frequencies, in individuals with vitiligo.

Regarding the precise impact of vitiligo on hearing thresholds, there are several disparities in different studies. Certain studies 10 14 17 21 22 23 claim that vitiligo affects hearing, whereas other studies 24 25 cast doubt on this claim. This dispute may arise from disparities in the selection criteria of subjects and the application of various methodologies in reaching a decision.

The abnormalities in the ABR recordings found in the present study included increased interpeak latency of waves II to III and divergence from normal values in wave I. Wave III is generally linked to activity mostly originating in the superior olivary complex (SOC) of the brainstem. The increased interpeak latency between waves I and III is attributed to disrupted synaptic activity and impaired transmission of action potentials along the auditory nerve to the lower brainstem. 14 25 Elsaied et al. 25 showed an increase in the interpeak latency of waves I to III in vitiligo patients, which was statistically significant and consistent with the findings of the current study.

## Limitations

Because the present study is observational and hospital-based, certain characteristics may not be accurately represented. Together with the sample size, there is still an additional restriction of not screening for related autoimmune or other systemic illnesses.

## Conclusion

To summarize, the evidence we have shown suggests that the melanocytes found in the auditory apparatus get involved in vitiligo, suggesting that the disease is likely to affect melanocytes throughout the body. Therefore, pure tone audiometry and ABR tests should be used to evaluate vitiligo patients even in the absence of hearing loss. An extensive auditory evaluation of these patients requires more research with larger samples and additional testing, including electrocochleography (EcochG) and otoacoustic emission (OAE) measurements.
